# A 44-Year-Old Man With a Solitary, Slowly Enlarging Mediastinal Lesion

**DOI:** 10.1016/j.chest.2025.04.037

**Published:** 2025-10-08

**Authors:** Vasiliki Kolokotroni, Garyfallia Perlepe, Angeliki Cheva, Christoforos Foroulis, Konstantinos I. Gourgoulianis

**Affiliations:** a Department of Respiratory Medicine, Faculty of Medicine, School of Health Sciences, University of Thessaly, Larissa, Greece; b Pathology Department, School of Medicine, Aristotle University of Thessaloniki, Thessaloniki, Greece; c Cardiothoracic Surgery Department, School of Medicine, Aristotle University of Thessaloniki, Thessaloniki, Greece

## Abstract

A 44-year-old man, who does not smoke, presented for investigation of a right mediastinum lesion as revealed on a routine radiograph that was performed as a requirement of his life insurance. The patient reported no associated symptoms. He did not report shortness of breath, cough, sputum, fever, chest pain, or hemoptysis. The remaining review of symptoms was also negative for issues such as muscle weakness, weight loss, night sweats, fatigue, skin rash, and visible, palpable, or painful lymphadenopathy. His medical history was unremarkable, and he did not receive any regular medication. The patient’s professional occupation was not related to special exposure and he did not report alcohol consumption, illicit drug use, or any recent travel.

## Physical Examination Findings

The patient was afebrile with stable vital signs: BP, 120/80 mm Hg; heart rate, 80 beats/min; oxygen saturation, 98% on ambient air; and respiratory rate, 13 breaths/min. Breathing was normal without accessory muscle use, cyanosis, or clubbing. Lung examination indicated symmetrical expansion, normal vocal vibration transmission, resonant percussion, and no abnormal sounds on auscultation. Cardiac assessment showed no apex displacement, murmurs, or thrills, and there was no jugular vein distention or peripheral edema. Ear, nose, and throat examination results were normal, with no signs of edema, hemorrhage, or abnormalities. Peripheral lymph nodes were unremarkable, and the remainder of the physical examination was normal.

## Diagnostic Studies

**Figure 1:**
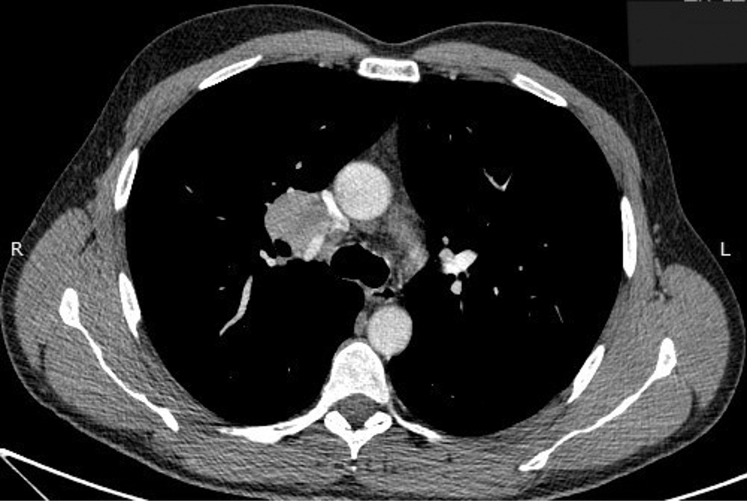
PET-CT scan showing a mildly hypermetabolic lesion at the right pulmonary hilum (standardized uptake value = 4.1).

**Figure 2:**
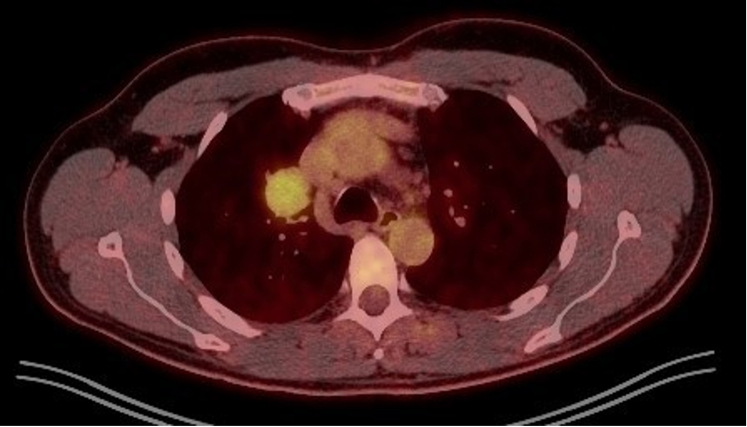
CT scan with contrast demonstrating a mild increase in the size of the lesion (3.7 × 3.4 × 3.1 cm).

The patient’s laboratory investigation results, including CBC and liver and renal function tests, were within normal ranges. Pulmonary function tests were also performed without impairment. The patient exhibited normal cardiac function, determined by a cardiologic assessment, accompanied by a sinus rhythm ECG and a triplex without abnormalities. Thereafter, further imaging with a non-contrast CT scan featured a solid space-occupying lesion at the right hilum measuring 3.5 × 3.1 × 3.0 cm, clearly defined, causing pressure on the superior vena cava. The next diagnostic step was a PET/CT, which highlighted a mildly hypermetabolic lesion at the right pulmonary hilum (standardized uptake value = 4.1) (Fig 1) as well as mild hypermetabolic activity at the gastric dome (standardized uptake value = 5.6), which was later diagnosed as gastritis through gastroscopy. The patient underwent an endobronchial ultrasound (EBUS), and the right paratracheal lymph node (4R), the right interlobar lymph node (11R), and the lesion were sampled. Cytology tests were not favorable for malignancy or granulomatous inflammation, and acid-fast stain and culture for *Mycobacterium* species were negative. A follow-up CT scan conducted after 4 months showed an increase in the size of the initial lesion (3.7 × 3.4 × 3.1 cm) (Fig 2).

**Figure 3:**
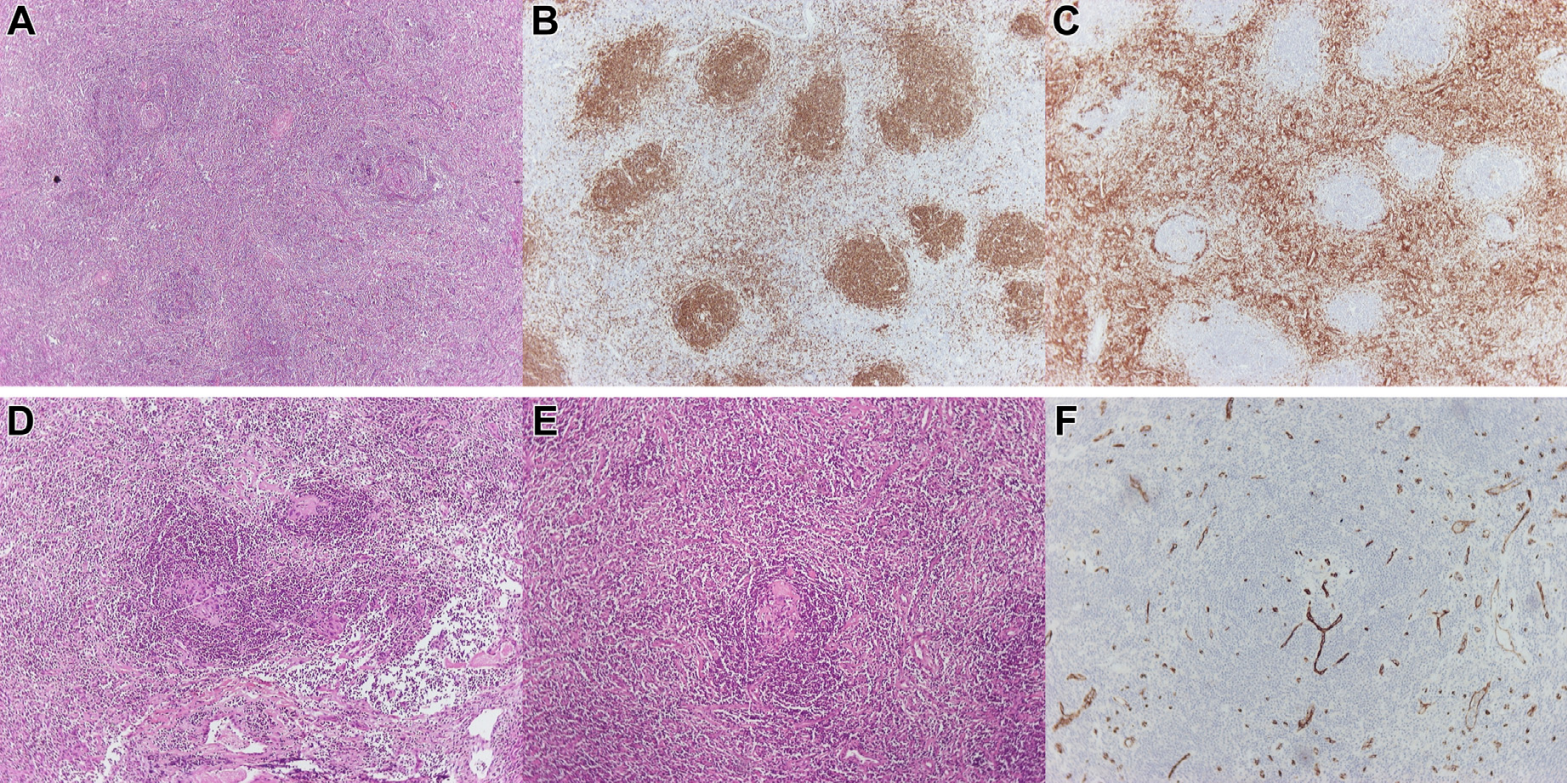
A, Hematoxylin and eosin staining: A lymph node with lymphoid follicles of small and large size; B, CD20: Marker for B-lymphocytes; C, CD3: Marker for T-lymphocytes; D, E, Hematoxylin and eosin staining: The vessels appeared hyalinized, and they created the characteristic ‘‘lollipop’’ appearance; F, CD34: Endothelial cell marker, highlights the blood vessels.

In accordance with this finding, the patient underwent a right thoracotomy and excision of the lesion at the right pulmonary hilum. Ηistopathologic evaluation showed a lymph node with small and large lymphoid follicles, prominent germinal centers, preservation of the dendritic cell network, and a concentric arrangement of mantle cells resembling “onion skin.” Additionally, the vessels appeared hyalinized, and they created the characteristic “lollipop” appearance (Fig 3). Limited plasma cell population revealed by plasma cell markers and staining for human herpes virus 8 (HHV8) showed no positive cells.

*What is the diagnosis?*

*Diagnosis:* Unicentric Castleman disease, hyaline-vascular type.

## Discussion

Castleman disease (CD) is a heterogeneous group of lymphoproliferative disorders that share common morphologic characteristics in lymph node biopsy. Based on the clinical presentation and disease course, CD is divided into unicentric CD (UCD), a localized disease involving a single enlarged lymph node or region of lymph nodes, and multicentric CD (MCD), a systemic, progressive, and often fatal disease characterized by lymphadenopathy in multiple lymph node stations. The Collaborative Network proposed a classification system further subdividing MCD based on its etiologic driver: HHV8-associated MCD (HHV8-MCD), POEMS-associated MCD (polyneuropathy, organomegaly, endocrinopathy, monoclonal plasma cell disorder, skin changes—POEMS syndrome), and idiopathic MCD (iMCD). Additionally, iMCD is further classified by phenotype into iMCD—TAFRO (co-incidence of thrombocytopenia, ascites, reticulin fibrosis, renal dysfunction, organomegaly) and iMCD—not otherwise specified (iMCD-NOS).

UCD is often found incidentally during the fourth decade of life. Symptoms, when present, are usually explained by the location or compression of not only nerves or blood vessels but also airways or ureters, whereas systemic symptoms such as weight loss, fever, or fatigue occur in only 10% to 20% of cases. Although all lymph node areas of the body can be affected, UCD tends to present as a single mass in the mediastinum. Little is known about the factors that may cause UCD. In several cases of UCD, clonal expansions of stromal cells have been identified, specifically follicular dendritic cells.

Regarding the treatment of UCD, surgical excision is the first-line therapy. Preoperative embolization of the lesion has often been tested to make the surgery safer. If surgery is not feasible or is too risky, further treatment depends on the presence of symptoms. Asymptomatic nonresectable UCD requires careful monitoring and regular follow-up with CT scans every 12 months. For symptomatic nonresectable UCD, there are several therapeutic approaches, such as partial excision or rituximab with or without corticosteroids, to reduce the size of the compressing lymph node. Those who remain symptomatic are candidates for radiation therapy.

CT scans of the neck, chest, and abdomen/pelvis should be used to differentiate UCD from MCD. PET-CT is also quite helpful because it reveals lymphadenopathy with primarily peripheral distribution and relatively symmetric and moderately hypermetabolic structures.

Histopathologic examination is essential for the definitive diagnosis of CD. EBUS bronchoscopy sampling is usually insufficient to establish CD, and patients undergo an open lung biopsy. Bronchoscopy is useful foremost to exclude other possible diagnoses, mainly metastatic neoplasms and sarcoidosis. Histologically, UCD is classified into the hyaline-vascular type, the plasma cell type, and an intermediate “mixed” type. Notably, the histologic characteristics exist along a continuum, rather than neatly falling into 3 distinct and well-defined categories. Hyaline-vascular type is more common, representing 91% of total CD cases. It is characterized by follicles with expanded mantle zones of small lymphocytes. The lymphocytes in the mantle zone are arranged concentrically, presenting a target-like pattern with a broad zone of small, mature lymphocytes that have condensed chromatin and minimal cytoplasm, giving an appearance like “onion skin.” Often, there may be radially invasive sclerotic blood vessels that, along with atrophic follicles and the concentric mantle zones, give the “lollipop” appearance. It is noteworthy that many other diseases exhibiting common histologic features with CD, such as autoimmune conditions, sarcoidosis, malignancies, and infections, must be excluded.

The response to treatment should be documented 1 to 3 months after the initial therapy through history, physical and laboratory examinations, and imaging findings. After excision, patients are followed up annually with CT scans and laboratory examinations. Annual imaging may be discontinued after 5 years, provided the patient remains disease-free.

### Clinical Course

In this patient who presented with a solitary mediastinal mass, revealed on a routine imaging study, malignancy had to be ruled out. Lack of fluorodeoxyglucose-avidity on PET scan, the small increase of the lesion in 4 months, and the negative results of EBUS for malignancy and granulomatous inflammation suggested against a diagnosis of malignancy. Given the fact that a hematologic disease was considered more likely, the patient underwent a thoracotomy for the mass to be removed. Diagnosis of UCD was confirmed by the histologic examination. The patient has been referred to the hematology department and will be monitored for a long period to rule out disease recurrence.

## Clinical Pearls

1. *UCD is generally characterized by asymptomatic slow growth of a lymph node station often located in the mediastinum*.
2. *For the diagnosis of UCD, an EBUS bronchoscopy is insufficient, and excisional biopsy is typically required*.
3. *High-contrast CT scan and PET-CT scan are important for ruling out MCD. Typical imaging findings are slow, progressive increase in the size of the mass and a subnormal SUV*.
4. *The treatment of choice for UCD is surgical excision. Continuous monitoring is essential to rule out recurrence of the disease*.

## Financial/Nonfinancial Disclosures

None declared.
